# Erratum

**DOI:** 10.1016/j.cjcpc.2023.05.002

**Published:** 2023-05-05

The publisher regrets that patient consent statements were not included in the published version of the following articles that appeared in previous issues of *CJC Pediatric and Congenital Heart Disease*.

The appropriate patient consent statements are included below.

1. “Medical Management of Infants with Supraventricular Tachycardia: Results from a Registry and Review of the Literature” [CJC Pediatric and Congenital Heart Disease, Volume 1, Issue 1, 2022, Pages 11-22] https://doi.org/10.1016/j.cjcpc.2021.09.001

**Patient Consent**

The authors confirm that a patient consent form(s) has been obtained for this article.

2. “Rare case of an adult with double chambered left ventricle” [CJC Pediatric and Congenital Heart Disease, Volume 1, Issue 1, 2022, Pages 37-39] https://doi.org/10.1016/j.cjcpc.2021.10.001

**Patient Consent**

The authors confirm that a patient consent form(s) has been obtained for this article.

3. “Canadian Developmental Follow-Up Practices in Children with Congenital Heart Defects: A National Environmental Scan” [CJC Pediatric and Congenital Heart Disease, Volume 1, Issue 1, 2022, Pages 3-10] https://doi.org/10.1016/j.cjcpc.2021.11.002

**Patient Consent**

The authors confirm that a patient consent form(s) has been obtained for this article.

4. “Prognostic Implications of Right Atrial Dysfunction in Adults with Pulmonary Atresia and Intact Ventricular Septum” [CJC Pediatric and Congenital Heart Disease, Volume 1, Issue 1, 2022, Pages 23-29] https://doi.org/10.1016/j.cjcpc.2021.11.001

**Patient Consent**

The authors confirm that patient consent is not applicable to this article.

5. “Massive Saddle Pulmonary Embolism in a Preterm Neonate with Successful Emergent Open Embolectomy” [CJC Pediatric and Congenital Heart Disease, Volume 1, Issue 1, 2022, Pages 40-43] https://doi.org/10.1016/j.cjcpc.2022.01.001

**Patient Consent**

The authors confirm that patient consent is not applicable to this article.

6. “Review of MIS-C Clinical Protocols and Diagnostic Pathways: Towards a Consensus Algorithm” [CJC Pediatric and Congenital Heart Disease, Volume 1, Issue 2, 2022, Pages 86-93] https://doi.org/10.1016/j.cjcpc.2022.01.003

**Patient Consent**

The authors confirm that patient consent is not applicable to this article.

7. “Sudden Cardiac Arrest in the Pediatric Population” [CJC Pediatric and Congenital Heart Disease, Volume 1, Issue 2, 2022, Pages 45-59] https://doi.org/10.1016/j.cjcpc.2022.02.001

**Patient Consent**

The authors confirm that patient consent is not applicable to this article.

8. “A Survey of Immunization Practices in Patients with Congenital Heart Disease” [CJC Pediatric and Congenital Heart Disease, Volume 1, Issue 2, 2022, Pages 74-79] https://doi.org/10.1016/j.cjcpc.2021.12.003

**Patient Consent**

The authors confirm that patient consent is not applicable to this article.

9. “Physical Activity in Pediatric Long QT Syndrome Patients” [CJC Pediatric and Congenital Heart Disease, Volume 1, Issue 2, 2022, Pages 80-85] https://doi.org/10.1016/j.cjcpc.2021.12.001

**Patient Consent**

The authors confirm that a patient consent form(s) has been obtained for this article.

10. “Double Trouble: Ductal Origin of Right Pulmonary Artery With Contralateral Embolism” [CJC Pediatric and Congenital Heart Disease, Volume 1, Issue 2, 2022, Pages 98-100] https://doi.org/10.1016/j.cjcpc.2022.02.002

**Patient Consent**

The authors confirm that a patient consent form(s) has been obtained for this article.

11. “The critical transfer from pediatrics to adult care in patients with congenital heart disease: Predictors of transfer and retention of care” [CJC Pediatric and Congenital Heart Disease, Volume 1, Issue 3, 2022, Pages 129-135] https://doi.org/10.1016/j.cjcpc.2022.04.003

**Patient Consent**

The authors confirm that patient consent is not applicable to this article.

12. “Cardiac Resynchronization Therapy Using Single-Site Left Ventricular Pacing in a Tricuspid Atresia Patient with Left Bundle Branch Block” [CJC Pediatric and Congenital Heart Disease, Volume 1, Issue 2, 2022, Pages 94-97] https://doi.org/10.1016/j.cjcpc.2022.01.004

**Patient Consent**

The authors confirm that a patient consent form(s) has been obtained for this article.

13. “Reducing barriers to optimal Automated External Defibrillator use: an elementary school intervention study” [CJC Pediatric and Congenital Heart Disease, Volume 1, Issue 1, 2022, Pages 30-36] https://doi.org/10.1016/j.cjcpc.2021.12.002

**Patient Consent**

The authors confirm that a patient consent form(s) has been obtained for this article.

14. “Approach to Wide Complex Tachycardia in Pediatric Patients” [CJC Pediatric and Congenital Heart Disease, Volume 1, Issue 2, 2022, Pages 60-73] https://doi.org/10.1016/j.cjcpc.2022.02.003

**Patient Consent**

The authors confirm that patient consent is not applicable to this article.

15. “Prospective assessment of coronary artery flows pre-and post-cardiopulmonary bypass in children with a spectrum of congenital heart disease” [CJC Pediatric and Congenital Heart Disease, Volume 1, Issue 3, 2022, Pages 119-128] https://doi.org/10.1016/j.cjcpc.2022.04.001

**Patient Consent**

The authors confirm that a patient consent form(s) has been obtained for this article.

16. “Early echocardiography predicts intervention need in antenatal suspicion of coarctation of the aorta” [CJC Pediatric and Congenital Heart Disease, Volume 1, Issue 4, 2022, Pages 167-173] https://doi.org/10.1016/j.cjcpc.2022.05.003

**Patient Consent**

The authors confirm that patient consent is not applicable to this article.

17. “Using Smartphone Wireless ECG Monitoring to Provide Symptom-Rhythm Correlation in the Pediatric Population” [CJC Pediatric and Congenital Heart Disease, Volume 1, Issue 3, 2022, Pages 158-161] https://doi.org/10.1016/j.cjcpc.2022.04.002

**Patient Consent**

The authors confirm that a patient consent form(s) has been obtained for this article.

18. “Pre-operative evaluation of refugee children with heart disease” [CJC Pediatric and Congenital Heart Disease, Volume 1, Issue 5, 2022, Pages 226-228] https://doi.org/10.1016/j.cjcpc.2022.05.004

**Patient Consent**

The authors confirm that patient consent is not applicable to this article.

19. “Exercise Capacity and Training Programs in Pediatric Fontan Patients: A Systematic Review” [CJC Pediatric and Congenital Heart Disease, Volume 1, Issue 3, 2022, Pages 108-118] https://doi.org/10.1016/j.cjcpc.2022.04.005

**Patient Consent**

The authors confirm that patient consent is not applicable to this article.

20. “A case of NAA10-related syndrome with prolonged QTc treated with a subcutaneous implantable cardioverter defibrillator after ventricular fibrillation” [CJC Pediatric and Congenital Heart Disease, Volume 1, Issue 6, 2022, Pages 270-273] https://doi.org/10.1016/j.cjcpc.2022.10.001

**Patient Consent**

The authors confirm that a patient consent form(s) has been obtained for this article.

21. “Determinants of Aortic Stenosis Progression in Bicuspid and Tricuspid Aortic Valves” [CJC Pediatric and Congenital Heart Disease, Volume 1, Issue 4, 2022, Pages 184-192] https://doi.org/10.1016/j.cjcpc.2022.06.004

**Patient Consent**

The authors confirm that a patient consent form(s) has been obtained for this article.

22. “Echocardiographic Assessment of Right Ventricular Function in Pediatric Heart Disease: A Practical Clinical Approach” [CJC Pediatric and Congenital Heart Disease, Volume 1, Issue 3, 2022, Pages 136-157] https://doi.org/10.1016/j.cjcpc.2022.05.002

**Patient Consent**

The authors confirm that patient consent is not applicable to this article.

23. “A cardiac arrest case due to left coronary artery compression in congenital heart disease-associated pulmonary arterial hypertension” [CJC Pediatric and Congenital Heart Disease, Volume 1, Issue 5, 2022, Pages 229-231] https://doi.org/10.1016/j.cjcpc.2022.04.004

**Patient Consent**

The authors confirm that a patient consent form(s) has been obtained for this article.

24. “Association of Acute Anti-Inflammatory Treatment with Medium-Term Outcomes for Coronary Artery Aneurysms in Kawasaki Disease” [CJC Pediatric and Congenital Heart Disease, Volume 1, Issue 4, 2022, Pages 174-183] https://doi.org/10.1016/j.cjcpc.2022.05.007

**Patient Consent**

The authors confirm that patient consent is not applicable to this article.

25. “Single-Center Case Series Assessment of Early Exercise Capacity Data among Patients Who Received an Alterra Prestent and SAPIEN 3 Valve Placement” [CJC Pediatric and Congenital Heart Disease, Volume 1, Issue 4, 2022, Pages 193-197] https://doi.org/10.1016/j.cjcpc.2022.06.002

**Patient Consent**

The authors confirm that patient consent is not applicable to this article.

26. “Reduced physical activity during COVID-19 in children with congenital heart disease: A longitudinal analysis” [CJC Pediatric and Congenital Heart Disease, Volume 1, Issue 5, 2022, Pages 219-225] https://doi.org/10.1016/j.cjcpc.2022.05.006

**Patient Consent**

The authors confirm that a patient consent form(s) has been obtained for this article.

27. “Reduction of atrial septal defect size after catheter ablation for atrial tachyarrhythmia and its predictive factors” [CJC Pediatric and Congenital Heart Disease, Volume 1, Issue 5, 2022, Pages 241-244] https://doi.org/10.1016/j.cjcpc.2022.08.001

**Patient Consent**

The authors confirm that a patient consent form(s) has been obtained for this article.

28. “Pentalogy of Cantrell” [CJC Pediatric and Congenital Heart Disease, Volume 1, Issue 4, 2022, Pages 198-199] https://doi.org/10.1016/j.cjcpc.2022.06.003

**Patient Consent**

The authors confirm that a patient consent form(s) has been obtained for this article.

29. “Aortic-valve sparing repair and homograft and Dacron pulmonary reconstruction long-term after Arterial Switch Operation” [CJC Pediatric and Congenital Heart Disease, Volume 1, Issue 4, 2022, Pages 200-202] https://doi.org/10.1016/j.cjcpc.2022.06.001

**Patient Consent**

The authors confirm that a patient consent form(s) has been obtained for this article.

30. “Arrhythmic burden of adult survivors with repaired total anomalous pulmonary venous connection” [CJC Pediatric and Congenital Heart Disease, Volume 1, Issue 6, 2022, Pages 263-269] https://doi.org/10.1016/j.cjcpc.2022.08.003

**Patient Consent**

The authors confirm that patient consent is not applicable to this article.

31. “Some things change, some things stay the same: Trends in Canadian education in pediatric cardiology and the cardiac sciences” [CJC Pediatric and Congenital Heart Disease, Volume 1, Issue 5, 2022, Pages 232-240] https://doi.org/10.1016/j.cjcpc.2022.08.004

**Patient Consent**

The authors confirm that patient consent is not applicable to this article.

32. “Pulmonary Hypertension in Children is associated with Abnormal Flow patterns in the Main Pulmonary Artery as demonstrated by Blood Speckle Tracking” [CJC Pediatric and Congenital Heart Disease, Volume 1, Issue 5, 2022, Pages 213-218] https://doi.org/10.1016/j.cjcpc.2022.09.001

**Patient Consent**

The authors confirm that patient consent is not applicable to this article.

33. “Effects of 12-Week Home-Based Resistance Training on Peripheral Muscle Oxygenation in Children with Congenital Heart Disease: A CHAMPS Study” [CJC Pediatric and Congenital Heart Disease, Volume 1, Issue 5, 2022, Pages 203-212] https://doi.org/10.1016/j.cjcpc.2022.08.002

**Patient Consent**

The authors confirm that a patient consent form(s) has been obtained for this article.

34. “Caring for the Aging Patient With Adult Congenital Heart Disease: A Review of Cardiac and Noncardiac Comorbidities” [CJC Pediatric and Congenital Heart Disease, Volume 1, Issue 6, 2022, Pages 274-281] https://doi.org/10.1016/j.cjcpc.2022.10.002

**Patient Consent**

The authors confirm that a patient consent form(s) has been obtained for this article.

35. “Balloon Atrial Septostomy: Does the balloon size matter?” [CJC Pediatric and Congenital Heart Disease, Volume 1, Issue 6, 2022, Pages 253-259] https://doi.org/10.1016/j.cjcpc.2022.10.006

**Patient Consent**

The authors confirm that patient consent is not applicable to this article.

36. “Automatic prediction of pediatric cardiac output from echocardiograms using deep learning models” [CJC Pediatric and Congenital Heart Disease, Volume 2, Issue 1, 2022, Pages 12-19] https://doi.org/10.1016/j.cjcpc.2022.11.001

**Patient Consent**

The authors confirm that patient consent is not applicable to this article.

37. “Observation of atrial fibrillation dependent on an intra-atrial reentrant tachycardia within the right atrium in a repaired tetralogy of Fallot” [CJC Pediatric and Congenital Heart Disease, Volume 2, Issue 1, 2022, Pages 51-54] https://doi.org/10.1016/j.cjcpc.2022.10.004

**Patient Consent**

The authors confirm that a patient consent form(s) has been obtained for this article.

38. “Socioeconomic status and Kawasaki disease outcomes in a single payer health care system” [CJC Pediatric and Congenital Heart Disease, Volume 1, Issue 6, 2022, Pages 248-252] https://doi.org/10.1016/j.cjcpc.2022.10.007

**Patient Consent**

The authors confirm that patient consent is not applicable to this article.

39. “The impact of COVID-19 on the cardiovascular health of emerging adults aged 18-25: findings from a scoping review” [CJC Pediatric and Congenital Heart Disease, Volume 2, Issue 1, 2022, Pages 33-50] https://doi.org/10.1016/j.cjcpc.2022.11.005

**Patient Consent**

The authors confirm that patient consent is not applicable to this article.

40. “Factors associated with acute kidney injury after cardiopulmonary bypass in children” [CJC Pediatric and Congenital Heart Disease, Volume 2, Issue 1, 2022, Pages 20-29] https://doi.org/10.1016/j.cjcpc.2022.11.007

**Patient Consent**

The authors confirm that a patient consent form(s) has been obtained for this article.

41. “Identifying predictors of psychological problems among adolescents with congenital heart disease for referral to psychological care: A pilot study” [CJC Pediatric and Congenital Heart Disease, Volume 2, Issue 1, 2022, Pages 3-11] https://doi.org/10.1016/j.cjcpc.2022.12.001

**Patient Consent**

The authors confirm that patient consent is not applicable to this article.

